# Economic evaluations of health technologies in Dutch healthcare decision-making: a qualitative study of the current and potential use, barriers, and facilitators

**DOI:** 10.1186/s12913-017-1986-9

**Published:** 2017-01-26

**Authors:** Kitty J. Roseboom, Johanna M. van Dongen, Emile Tompa, Maurits W. van Tulder, Judith E. Bosmans

**Affiliations:** 1 Department of Health Sciences and the EMGO+ Institute for Health and Care Research, Faculty of Earth and Life Sciences, VU University Amsterdam, De Boelelaan 1085, 1081 HV Amsterdam, The Netherlands; 2 Institute for Work & Health, 481 University Avenue Suite 800, Toronto, ON M5G 2E9 Canada; 3 Department of Economics, McMaster University, Kenneth Taylor Hall Room 426, 1280 Main Street West, Hamilton, ON L8S 4 M4 Canada; 4 Dalla Lana School of Public Health, University of Toronto, 155 College Street Health Science Building, 6th floor, Toronto, ON M5T 3 M7 Canada

**Keywords:** Economic evaluation, Barriers and facilitators, Use, Healthcare decision-making

## Abstract

**Background:**

The use of economic evaluations in healthcare decision-making can potentially help decision-makers in allocating scarce resources as efficiently as possible. Over a decade ago, the use of such studies was found to be limited in Dutch healthcare decision-making, but their current use is unknown. Therefore, this study aimed to provide insight into the current and potential use of economic evaluations in Dutch healthcare decision-making and to identify barriers and facilitators to the use of such studies.

**Methods:**

Interviews containing semi-structured and structured questions were conducted among Dutch healthcare decision-makers. Participants were purposefully selected and special efforts were made to include decision-makers working at the macro- (national), meso- (local/regional), and micro-level (patient setting). During the interviews, a topic list was used that was based on the research questions and a literature search, and was developed in consultation with the Dutch National Healthcare Institute. Responses to the semi-structured questions were analyzed using a constant comparative approach. As for the structured questions, participants’ definitions of various economic evaluation concepts were scored as either being “correct” or “incorrect” by two researchers, and summary statistics were prepared.

**Results:**

Sixteen healthcare decision-makers were interviewed and two health economists. Decision-makers’ knowledge of economic evaluations was only modest, and their current use appeared to be limited. Nonetheless, decision-makers recognized the importance of economic evaluations and saw several opportunities for extending their use at the macro- and meso-level, but not at the micro-level. The disparity between the limited use and recognition of the importance of economic evaluations is likely due to the many barriers decision-makers experience preventing their use (e.g. lack of resources, lack of formal willingness-to-pay threshold). Possible facilitators for extending the use of economic evaluations include, amongst others, educating decision-makers and the general population about economic evaluations and presenting economic evaluation results in a clearer and more understandable way.

**Conclusions:**

This study demonstrated that the current use and impact of economic evaluations in Dutch healthcare decision-making is limited at best. Therefore, strategies are needed to overcome the barriers that currently prevent economic evaluations from being used extensively.

**Electronic supplementary material:**

The online version of this article (doi:10.1186/s12913-017-1986-9) contains supplementary material, which is available to authorized users.

## Background

As in other developed countries, a large proportion of the Dutch Gross Domestic Product is spent on healthcare, and this proportion has grown during the last decade, from 9% in 2004 to 11% in 2014 [1]. It has been suggested that such increases can be explained by factors outside the healthcare sector (e.g. ageing population, increased prevalence of chronic diseases), the absence of a competitive market within healthcare systems, the absence of strong cost-containment measures, and technological innovation [2, 3]. Of them, technological innovation is often cited as the main driver of the long-term growth in healthcare costs [3–5]. It must be noted that high and rising healthcare costs are not necessarily associated with negative connotations. Cost increases may be synonymous with improved health outcomes, increases in job opportunities in the sector, and improved quality of services delivered [2, 6]. However, since the rate of increase in healthcare costs currently exceeds economic growth, its continued growth at current rates is not sustainable and public spending on healthcare is crowding out public spending on other services. As a consequence, there is a strong (political) call for healthcare cost-containment and healthcare decision-makers are increasingly being confronted with choices about which treatments to reimburse and which to not reimburse [7].

Economic evaluations provide an indication of the relative efficiency of treatments by comparing the costs and consequences of alternative programs or interventions [8]. Such studies can help healthcare decision-makers to determine how best to allocate scarce resources at the macro-, meso-, and micro-level. At the macro-level, the Dutch Ministry of Health, Welfare, and Sports has to decide upon the content of the basic health insurance package (i.e. a compulsory insurance for all Dutch citizens). In making such decisions they are advised by the Dutch National Healthcare Institute, an independent governing body that, amongst others, provides evidence-based guidance and advice on the in- or exclusion of healthcare services in the basic health insurance package as well as the conduct of economic evaluations [9, 10]. The majority of the content of the basic health insurance package, however, is somewhat openly formulated [10]. This means that ‘insured care’ is defined in terms of functions of care rather than in specific healthcare services [10]. As a consequence, the responsibility for ‘appropriate use’ of insured care, and thus the allocation of the healthcare budget, is partly transferred to institutions and healthcare providers working at the regional or local level (i.e. meso-level) and in the individual patient setting (i.e. micro-level) [11].

Various studies indicated that healthcare decision-makers in many Western countries have a positive attitude towards the use of economic evaluations for resource allocation decision-making, but that their use and knowledge of economic evaluations is limited [12–17]. This discrepancy is likely due to the various barriers that decision-makers experience preventing their use in day-to-day decision-making, such as a lack of resources, political opposition, and a lack of relevant studies [12–17]. The only Dutch study to explore the use of economic evaluations in healthcare decision-making was performed more than a decade ago (i.e. 1998–1999) [17]. However, in an effort to improve the quality and efficiency of care, the Dutch government introduced a new Health Insurance Act in 2006, changing the healthcare system from a partly public and partly private, predominantly government-run system, into a universal insurance market that aims to be competitive [10]. Amongst others, the new act mandates all Dutch citizens to purchase the basic health insurance package, all insurance companies have to offer the basic health insurance package, and competing health insurance companies are obliged to accept all applicants during an annual enrollment period [10]. As one of the main aims of the Dutch healthcare reform was to improve the efficiency of healthcare [10, 18], it is conceivable that the decision-makers’ knowledge and use of economic evaluations have increased since then. Whether this is indeed the case, however, is currently unknown. Therefore, this study aimed to gain insight into the current and potential use of economic evaluations in Dutch healthcare decision-making and to identify barriers and facilitators to the use of such studies.

## Methods

Interviews containing both semi-structured and structured questions were conducted among Dutch healthcare decision-makers to explore their economic evaluation knowledge and skill set, the current and potential use of economic evaluations in the healthcare decision-making context, as well as barriers and facilitators to the use of such studies. For the purpose of this study, “healthcare decision-maker” was defined as a professional who has influence on the allocation of the Dutch healthcare budget at the macro-, meso-, and/or micro-level. A predominantly qualitative approach was used in order to explore the questions under study in greater detail and to obtain in-depth information on the views and opinions of the participants [19].

### Study population

Participants were purposefully selected using a combination of critical case and maximum variation sampling, in which a small, but heterogeneous, sample of information-rich cases was selected [20, 21]. Special efforts were made to include decision-makers working at the macro-, meso-, and micro-level, and in different regions of the Netherlands. Additionally, two health economists with a deep understanding of the Dutch healthcare system were included. The Dutch National Healthcare Institute assisted in identifying participants for this study. Participants were also selected by means of snowballing, i.e. they were referred by other participants on the basis that they were expected to be able to provide relevant information [22]. Potential participants were contacted via email.

At the start of the interviews, all participants were informed about the study purpose, were reassured of confidentiality, and provided verbal informed consent. The present study was conducted in accordance with the Declaration of Helsinki. Under Dutch law, ethical approval was not necessary for this study, since it is not required for studies that do not infringe the participants’ physical and/or psychological integrity (according to the Dutch Medical Research Involving Human Subjects Act).

### Interviews

Interviews were conducted in Dutch and were carried out by one researcher (KR) between April and June 2014 at a time and location convenient to the participants. Participants were informed that they did not have to prepare for the interviews and were assured that there were no right or wrong answers. All interviews started with several short questions about the participants’ demographic and employment characteristics. Subsequently, semi-structured questions were asked about their current and potential use of economic evaluations for decision-making, as well as barriers and facilitators to their use. The topic list for the interviews was developed based on the research questions and a literature search in PubMed, the Cochrane-library, and Google Scholar. The literature search was aimed at getting a general overview of previous studies evaluating the use of economic evaluations in healthcare decision-making. The search was conducted by KR, with the help of an information specialist of the VU University Medical Centre. Search terms included: “economic evaluation”, “cost-effectiveness”, “cost-benefit analysis”, “decision-making”, “healthcare rationing”, “treatment decision”, “attitude”, “health personnel attitude”, “knowledge”, and “economic evaluation skills”. For completeness, reference lists of included studies were screened. Out of 44 publications, 15 articles were selected and studied in-depth by KR to formulate (sub-) topics. The topic list was further refined in consultation with two employees of the Dutch National Healthcare Institute. The final topic list can be found in Additional file 1.

During the interviews, the topic list was used as a guide, but participants were allowed to discuss other topics that they considered to be important as well. Throughout the interviews, participants were asked to clarify their answers by providing daily decision-making examples. To investigate the decision-makers’ knowledge of economic evaluations (excluding health economists), interviews ended with a number of structured questions. First, participants were asked what they associated with the term ‘economic evaluation’ and whether they had previously received training in economic evaluation-related topics. Subsequently, they were asked whether they were familiar with and could define various economic evaluation designs, including cost-effectiveness analysis (CEA), cost-benefit analysis (CBA), and cost-utility analysis (CUA). During the interviews, field notes were taken and interviews were audio-taped and transcribed verbatim directly after the interview. As no additional information emerged from the data after 18 interviews (i.e. data saturation was reached), the data collection process was terminated.

#### Data analysis

Using Nvivo 10, data derived from the semi-structured questions were analysed using a constant comparative approach. That is, analytic categories were inductively established by constantly comparing and checking items with the rest of the data [23]. By starting with open coding, descriptive themes and subthemes were generated by KR. That is, transcripts were read line by line and relevant passages were selected and coded. Throughout this process, efforts were made to detect further examples of previously identified (sub-) themes and, if applicable, to identify new ones. The final codes were developed through discussion between two researchers (KR and JvD). During these discussions, similar codes were grouped into analytical categories and the different properties of these categories were explored (i.e. the characteristics of these categories) as well as the relationships between them (i.e. relating categories to each other) [24, 25]. For the purpose of this article, quotes were translated from Dutch to English by the research team and were carefully edited slightly to make them more readable without losing their meaning.

Data derived from the structured-questions were analyzed using descriptive statistics. For this purpose, KR and JvD independently scored the participants’ definitions of CEA, CUA, and CBA as ‘correct’ or ‘incorrect’. Definitions were scored as ‘correct’ if they included some combination of the following information: CEA, a comparison of costs and effects, in which effects are expressed in terms of health effects (other than those expressed in terms of a quality of life measure or a monetary value); CUA, a comparison of costs and effects, where effects are expressed in QALYs (quality adjusted life years) or an appropriate variant taking quality of life into account; CBA, a comparison of costs and benefits, where the benefits get a monetary value [25]. In all other cases, they were scored as ‘incorrect’. After both researchers independently scored the definitions, scores were compared and disagreements (*n* = 6) were resolved through discussion between KR and JvD [25].

## Results

### Participants

In total, 20 potential participants were approached, of whom two declined to participate; one due to time constraints and one considered him/herself not suitable for this study. Eventually, seventeen interviews were conducted face-to-face and one participant answered the questions by email. Interviews (excluding email contact) lasted on average 49 min [range:31–72 min]. Twelve participants were male (66.7%). Participants had a mean age of 49.7 years (SD = 8.4). Sixteen participants were healthcare decision-makers working at the macro- (*n* = 5; labelled as MA1-5), meso- (*n* = 4; labelled as ME1-4), or micro-level (*n* = 7; labelled as MI1-7), and two were health economists (labelled as HE1-2). Macro-level decision-makers worked at the Dutch National Healthcare Institute (*n* = 4) or the Dutch Ministry of Health, Welfare and Sports (*n* = 1). Meso-level decision-makers worked for a health insurance company (*n* = 2) or as a guideline development consultant (*n* = 2). All micro-level decision-makers were physicians (*n* = 7). Participants had various educational backgrounds, but mostly medical (55.6%) and economic (27.8%) (Table 1).

**Table 1:** Baseline characteristics of the study population

Baseline characteristics	Total *n* = 18
Male [*n* (%)]	12 (66.7)
Age (years) [mean (SD)]	49.7 (8.4)
Educational training [*n* (%)]	
Medical	10 (55.6)
Economic	5 (27.8)
Medical + economic	1 (5.6)
Remaining	2 (11.1)
Working level [n (%)]	
Macro-level	5 (27.8)
Meso-level	4 (22.2)
Micro-level	7 (38.9)
Health economists	2 (11.1)

Note: Macro-level: decision-makers working at the national level; Meso-level: decision-makers working at the regional or local level; Micro-level: decision-makers working in the individual patient setting

### Results of the structured interview questions

#### Knowledge of economic evaluations

While 63% of the participants working at the macro- and meso-level (excluding health economists) indicated that they had received some training in economic evaluation-related topics, none of those working at the micro-level had received such training.

When asked about what they associated with the term ‘economic evaluation’, participants frequently thought it to be solely a calculation of costs and many were not aware of the existence of various kinds of economic evaluations. *“I think that this would provide a proper overview of all costs. (…) Possibly, also compare those costs with one another, but in my opinion an economic evaluation does not amount to much more than that. I wouldn't actually correlate it with effectiveness.” (ME4)* Of the participants, 36% were able to give a correct definition of the concept of CEA. Other participants thought it to solely include costs or thought its outcome depended on a predetermined goal, as illustrated by the following quote; *“Cost-effectiveness of course really depends on the stated goal: when is a treatment effective? What is the objective you are aiming for?” (MI5)* Although 36% of participants were able to give a correct definition of the concept of CUA, many had never heard of the term, or thought it to be related to ‘utilization’. One participant, for example, responded; *“It seems to me more a technical term, frankly. (…) Utilization no, I don’t have an immediate explanation.” (MI5).* Most participants indicated familiarity with the concept of CBA, but only half was able to give a correct definition. Some participants thought it to be a broader concept compared to CEA, i.e. more costs and effects are taken into account. As one participant stated: *“And with CBA you weigh all the effects of the intervention. So also the effects on production loss. Well, all social effects, I think?" (MI4).*

### Results of the semi-structured interview questions

The (sub-) themes that emerged during the analysis of the current and potential use of economic evaluations for healthcare decision-making, as well as the experienced barriers and facilitators to the use of such studies will be discussed below, and will be illustrated by quotes.

#### Current use of economic evaluations

Participants generally agreed that there is a need for improving the efficiency of healthcare. The use of economic evaluations was therefore thought to be inevitable. One participant, for example, stated; *“I believe that a 30% cost increase took place there [in Mental Healthcare] in just a few short years (…) Well, in that case it's definitely worthwhile to pay attention to economic evaluations. If we allow costs to keep on rising, we will soon have no schools left and no asphalt on our roads.” (MA1)* Nonetheless*,* the current use and impact of economic evaluations in healthcare decision-making seemed to be limited. Participants generally indicated that economic evaluations were not a dominant factor in the decision-making process and that economic evaluations hardly ever impacted the inclusion or exclusion of a specific treatment in the basic health insurance package. One participant, for example, stated; *“Up until now, I have had to conclude that economic evaluations do not form the deciding factor, or hardly ever, in reaching a negative package advice. We always examine cost-effectiveness, but when matters come to a head, you realise that neither the government nor society is ready to make negative reimbursement decisions on cost-effectiveness results.” (MA2).*

Participants indicated that reimbursement and/or treatment decisions are typically based on the effectiveness of a treatment, rather than on its cost-effectiveness, as well as physicians’ desire to provide a certain treatment to their patients. Some participants attributed the limited use of economic evaluations to the fact that their necessity is not sufficiently recognized, both by healthcare decision-makers and the general population. As one health economist metaphorically stated; *“One way or another, it's as if the water has to rise even higher before we decide that we need to build dikes." (HE1).*

Even though the role of economic evaluations in healthcare decision-making was considered limited, some participants were able to provide examples of decision-making processes in which economic evaluations have been consulted. At the macro-level, for example, economic evaluations were used in the process of determining the content of the basic health insurance package, during price negotiations between the Dutch Ministry of Health, Welfare, and Sports and pharmaceutical companies, and during the implementation of a population wide screening tool. At the meso-level, economic evaluations have been used during the development of clinical guidelines and the implementation of innovations within healthcare organizations. Within healthcare organizations, however, only CBAs were used. Participants were not able to provide examples of the use of economic evaluations at the micro-level.

#### Potential use of economic evaluations

Participants provided various examples of decision-making processes during which the use of economic evaluations could prove to be beneficial. At the macro-level, the government could use economic evaluations to determine what expenditures, within the healthcare sector or even in other sectors, are likely to provide the best value for money. Furthermore, economic evaluations could be used during price negotiations between health insurers and healthcare providers (meso-level) as a means to generate the lowest healthcare prices possible. Other options for using economic evaluations at the macro-level include the narrowing of medical indications (e.g. defining patient groups for whom specific treatments are cost-effective and for whom they are not) and improvements in the organization of healthcare processes (e.g. decisions to shift certain treatments from secondary to primary care, and vice versa). Even though participants saw several opportunities for extending the use of economic evaluations at the macro- and meso-level, almost all agreed that there was no room for using economic evaluations in the individual patient setting (micro-level). One of their main arguments was that talking about costs would potentially disrupt the doctor-patient relationship. This is illustrated by the following quote; *“Patients become very suspicious when you start talking about costs. (…) In fact, it can stand in the way of a doctor-patient relationship.” (MI4)*. Some participants also emphasized that improving the efficiency of healthcare ought not to be the responsibility of the individual healthcare provider. As one health economist noted; *“In my opinion, the preconditions under which physicians work, that is, the financial framework that we succeed in creating with one another, are not the responsibility of individual doctors.” (HE1).*

#### Barriers to the use of economic evaluations

Participants identified various factors that currently prevent economic evaluations from being extensively used in healthcare decision-making.

##### Lack of resources:

All participants indicated that a lack of resources (i.e. time, money, skills) often prevent economic evaluations from being used in healthcare decision-making. As indicated by one participant: *“But the only thing the profession says is, bring on the money. (…) Money really is the only thing.” (ME4).*

##### Methodological factors:

Participants identified various kinds of barriers related to the methodology of economic evaluations. An important barrier was the way in which costs are typically valued in economic evaluations. Some participants pointed out that, while costs are often based on standard prices in economic evaluations, they may vary extensively in reality due to factors such as the jurisdiction in which a decision is made as well as the size and degree of specialisation of a healthcare facility. As one participant noted; *“But costs differ enormously across countries, due to differences in the cost of equipment, personnel etc. So it has to be determined per country separately. (…) Even within the Netherlands, the price of a day spent in a hospital differs across hospitals.” (MI5)* Many prices in the Dutch healthcare sector are set during negotiations between health insurers and healthcare providers, and can therefore differ enormously from standard prices. Likewise, economic evaluations are often conducted with only a couple of perspectives, such as the societal one that considers all costs and consequences including those outside of the healthcare sector. Consequently, many economic evaluations are not directly applicable to specific decision-making contexts. As one participant noted; *“An example of a concrete matter that we are struggling with is whether you should only include direct costs or also indirect costs. (…) We calculated that, in a given scenario, there will be a certain number of prescriptions and that this will lead to a certain reduction in complications and mortalities. If we would also include the prevention of mortalities, however, the cost-saving of €18 million would change in a cost-increase of about €240 million…” (ME3).* Another barrier is that healthcare decision-makers often poorly understand outcome measures used in economic evaluations. Participants had difficulties with interpreting QALYs, viewed them of limited value to long-term care, and were uncertain about their transferability across countries. As for their limited value to long-term care, one health economist stated *“The trade-off between curative and long-term care is always a theme, as you won’t get far using QALYs. Then you need something else and can you still weigh those results then?” (HE2)* Some participants were also concerned about the existence of interventions of which the results are difficult to measure. As indicated by one participant: *“In paediatrics, it is the professions whose effectiveness is not so obvious that we need most, such as psychologists, physiotherapists; the paramedics in fact. But I think it will be no easy task to demonstrate their cost-effectiveness.” (ME3).*

##### Lack of confidence in economic evaluations:

In the case of *model-based* economic evaluations, participants doubted their reliability due to the complexity of the healthcare system, which they considered to be hard to account for in a model. As one participant noted; *“A model is a simplification of reality. (…) I think that is where the problem in healthcare begins. There are so many influencing factors (…) making it very difficult to construct such a model.” (MA1).* Also, some participants indicated to lack confidence in economic evaluations in general. They attributed this to the fact that economic evaluations are sometimes funded or performed by the supplier of the treatment under study (i.e. there can be an inherent conflict of interest). This is illustrated by the following comment: *“I think there is also research showing that if a supplier initiates it [the study], the results often turn out more positive.” (MI3).*

##### Lack of a formal willingness-to-pay threshold:

Participants indicated that the lack of a formal willingness-to-pay threshold is an important barrier to the use of economic evaluations in healthcare decision-making. Therefore, most participants were in favour of establishing such threshold. As one participant noted; *“I find it inconsistent that we aren’t brave enough to talk about what we want to spend on the life of a human being in the Netherlands. What is a life-year gained allowed to cost?” (MI6)* Aside from the fact that the establishment of a formal willingness-to-pay threshold may help decision-makers in choosing between alternatives, it may also release physicians of the responsibility of incorporating efficiency considerations into the individual patient setting. This reasoning is substantiated by the following comment; *“I can quite understand that physicians say it is going too far to expect them to determine the limits (…) If you ask me, it is entirely reasonable that physicians need some help with this.” (MA1)* Although a formal willingness-to-pay threshold may improve the uptake of economic evaluations, participants generally agreed that it would be difficult to set such a threshold. Participants emphasized that when determining the maximum cost per additional unit of effect, many factors should be taken into account, including the prevalence and severity of a disease, the patients’ age, preferences, and prognosis, and the availability of alternative options. As one participant noted *“It matters a lot whether alternative treatment options are available. (…) If not, healthcare decisions concern questions about life and death”* (MA2). According to some participants, it is therefore unethical to use a fixed willingness-to-pay threshold, particularly when it concerns life-threatening diseases. As such, it was suggested by some participants that a willingness-to-pay threshold should be extendible or categorized. The few participants who disagreed with the need for a formal willingness-to-pay threshold did so because they thought that savings could still be made in other areas (e.g. by reducing the number of unnecessary diagnostic tests) or because they felt that they would lose some of their authority as physicians. Another participant feared that the introduction of a formal willingness-to-pay threshold would lead to future economic evaluations producing incremental cost-effectiveness ratios below the threshold, particularly in the case of modelling studies; *“I think models are easily influenced. And if you're going to choose a fixed threshold then you will see that the results of all models go toward that threshold.” (ME3).*Lack of relevant economic evaluations: Participants indicated a need for economic evaluations that are directly applicable to their decision-making context, whereas economic evaluations are often based on restrictive patient populations and/or settings, and are sometimes already outdated at the time of publication. As one participant indicated; *“An awful lot of economic evaluations are based on selective groups of patients (…) Generally, trial patients are not the same as those you encounter in daily practice” (MI1).*

##### Public resistance:

Participants indicated that for economic evaluations to be used in healthcare decision-making, it is essential that not only decision-makers, but also members of the general population understand, appreciate, and support their use. This is due to the Dutch healthcare system being largely publicly funded. Decisions made within this system therefore involve essentially the entire population, because everyone contributes to the financing of healthcare. Several participants, however, thought the general population is ‘not yet ready’ to accept efficiency considerations as a factor in healthcare decision-making. They attributed this to the fact that most people either misunderstand the purpose of economic evaluations or consider the idea of a maximum price for healthcare unacceptable, particularly when they are the ones being sick. One participant explained this as follows; *“When you have a problem, you want the best possible care. At such a moment, you are not interested in the macro-economic aspect that it will lead to enormous costs if it happens a thousand times.” (MI2)* Some participants also believed the general population’s knowledge of economic evaluations to be insufficient. As one participant noted; *“Total costs and cost-effectiveness are all jumbled up together and we have discovered …. [in a research project] that even the most well-educated people really do not know how it works.” (MA2)* Participants therefore believed that the general population needs to have some basic knowledge of (the purpose of) economic evaluations in order for them to be able to substantiate their acceptance or rejection of the use of economic evaluations. As indicated by one participant: *“The price of a drug is something patients can comprehend, whereas concepts such as QALYs are not. While, on the other hand, I think it is a good tool [economic evaluations], because people might think ‘my life has no price, I should get the best possible care’, but cost-effectiveness results are more nuanced in that the costs are weighed against the effectiveness of a drug” (MA3).*

##### Ambiguity among decision-makers about the physicians’ responsibility for improving the efficiency of healthcare:

Participants indicated that in order for economic evaluations to be used in healthcare decision-making, decision-makers themselves need to feel more responsibility for improving the efficiency of healthcare. However, since the main role of physicians is to act in a patient’s best interest, it is not self-evident that they can embrace such a responsibility. This is underscored by the following statement of a participating physician: *“I definitely want to keep it out of surgery. Because I find it very difficult if at a certain stage I have to make choices based on financial criteria only.” (MI3)* While participants working at the macro- and meso-level generally agreed that physicians should contribute to improving the efficiency of healthcare, physicians themselves did not agree on what this should entail. Some of them noted that the use of economic evaluations in the individual patient setting may raise ethical concerns, as it can appear to be in contradiction with the Hippocratic Oath. As one participant noted; *“Well, I think (…) naturally, we want to give patients what we think they need and this represents a big barrier if a cost-effectiveness analysis shows that it is actually too expensive, while we feel that the patient does have a right to it. (…) this is at odds with our oath.” (MI4)* The Hippocratic Oath reminds physicians of their social responsibility as well as their responsibility for the individual patient; i.e. two tasks that are often incompatible when costs are considered. This is illustrated by the fact that one participating physician stated; *“Up until a few years ago, the crux of the matter was in fact that you always gave people the care they needed and no-one even considered the cost. I think that, as physicians, we find this inversion extremely difficult, really I do.” (MI7),* whereas a decision-maker working at the meso-level argued; *“This is actually mentioned in the Hippocratic Oath (….) cost-effectiveness is a part of the oath that we all take. This is something that physicians sometimes forget (….)” (ME1).*

##### Incentives to treat:

Some participants regarded the abundance of ‘incentives to treat’ in the healthcare system as a barrier to the use of economic evaluations. As the Dutch healthcare system currently has a fee-for-service system, physicians and/or the organizations they work for, make money by treating patients, whereas abstention from treatment generates less income. Moreover, patients often prefer some treatment than no treatment. As a consequence, if physicians feel responsibility for improving the efficiency of healthcare, it is still difficult for them to act accordingly. As one participant noted; *“The human body is an inexhaustible source of income. As long as these stimuli exist, the problem will never be solved. There are simply financial stimuli for taking action, for doing something. The system does not have stimuli for making economic use of your resources” (MI1).*

##### Limited ability to shift resources across sectors:

Another barrier to the use of economic evaluations concerns the decision-makers’ limited ability to shift resources between and within sectors. To illustrate, if an economic evaluation shows that a specific treatment is more likely to be cost-effective if provided in primary care than in secondary care, it would be necessary to shift resources within the healthcare sector, but this is not easily executed. If an economic evaluation is performed from the societal perspective, savings flowing into non-healthcare sectors can lead to positive cost-effectiveness results, whereas the cost-effectiveness results for the healthcare sector itself may be unfavourable. The latter is illustrated by the following comment; *“How do you process, for instance, benefits that are accrued outside healthcare? (…) Because then the outcome of an analysis would be that everything [within the healthcare sector] would become much more expensive and that could hamper implementation, while it does actually lead to results, just not in healthcare.” (ME3).*

#### Facilitators for the use of economic evaluations

The most frequently mentioned facilitator for extending the use of economic evaluations was educating decision-makers about how to understand and interpret economic evaluations of health technologies and how to use them in resource allocation decision-making. Participants emphasized that some basic training about health economics, and economic evaluations in particular, should be included in the medical curriculum. One participant, for example, stated; *“This is an aspect of training that is completely neglected, even in medical follow-up training.” (MI1)* According to the participants, health economists could do their bit by presenting their results in a clearer and more understandable way. This is exemplified by the following comment; *“Well, I think that perhaps the language used by health economists should be more neutral, with more layman's terms, so that it is at least clearer; they should use plainer language, especially for those who are less well educated.” (MA3)* Participants also recognized the necessity of educating the general population about (the purpose of) economic evaluations in order to build the necessary public acceptance. As one participant noted; *“It is often very difficult to get such abstract ideas about cost-effectiveness across and it actually demands a lot of insight.” (MA2)* Moreover, financial and intellectual support was emphasized as a requirement for making the use of economic evaluations feasible and to create incentives for decision-makers to start using them. As one participant noted; *“You need support for cost-effectiveness analysis, because it is not so easy” (MI4)* Some participants were also of the opinion that the reliability, consistency, and transparency of economic evaluations themselves ought to be improved and that industry-funded studies should be assessed more critically. As one participant stated; *“I am actually in favour of spending more money on carrying out, or improving, economic evaluations, because this would result in less bias. Because currently many of them are carried out by private manufacturers, so from that point of view, you would like a more trustworthy assessment” (MA3).*

## Discussion

### Main findings

This study illustrates a need to advance Dutch healthcare decision-makers’ economic evaluation skill set, as their current knowledge of economic evaluations is quite modest. Moreover, even though participants were able to provide some examples of Dutch healthcare decisions in which economic evaluations were consulted, the current use and impact of such studies appeared to be limited at best. Nonetheless, decision-makers recognized the importance of economic evaluations and saw several opportunities for extending their use at the macro- and meso-level, but not at the micro-level. This disparity between the limited use of economic evaluations and the decision-makers’ recognition of their importance might be explained by the many barriers decision-makers experienced with their use (e.g. lack of required resources, lack of formal willingness-to-pay threshold).

### Comparison with the literature

The present findings are in line with those of previous studies demonstrating that the use and impact of economic evaluations of health technologies in healthcare decision-making is limited in most Western countries [12–17]. Also, in most of the previous studies comparable barriers and facilitators to the use of economic evaluations were identified [12]. The Dutch study that explored the use of economic evaluations in healthcare decision-making prior to the Dutch healthcare reform also found decision-makers to have a positive attitude towards economic evaluations, whereas their actual use and knowledge of such studies was limited [17]. The decision-makers participating in that study also indicated difficulties with moving resources across sectors, a lack of relevant studies, a lack of confidence in model-based and industry-sponsored economic evaluations, and a lack of economic evaluation knowledge to obstruct to the use of such studies in daily decision-making practice, whereas other barriers identified in the present study (e.g. lack of a formal willingness-to-pay threshold, public resistance) were not mentioned. In both studies, macro-, meso-, as well as micro-level decision-makers were included and semi-structured interviews were used for answering the research questions. All in all, this indicates that the use of economic evaluations has not increased extensively over the last decade, whereas there seem to have been some developments since then, with the most important one being the fact that the majority of the participants in the present study argued in favour of the establishment of a formal willingness-to-pay threshold, whereas participants in the previous study were generally against the introduction of such a threshold [17].

### Strengths and limitations

A strength of the present study is that it is qualitative. An important advantage of a qualitative design is that it allows for the questions being studied to be explored in greater detail and for the collection of in-depth information on participants’ views and opinions. The latter is particularly important because many of the previous studies in this area, and those conducted outside the Netherlands in particular, relied heavily on survey methods, which limited the participants’ freedom of response [12]. As such, the present findings are likely to be relevant to other jurisdictions as well, as many other countries are also trying to deal with high and rising healthcare costs by searching for means to maximize health effects within their fixed healthcare budget. Another strength concerns the fact that this was the first study since the Dutch healthcare reform in 2006 to explore the current and potential use of economic evaluations in healthcare decision-making. Given that the reform was inter alia aimed at improving the efficiency of healthcare, our study serves a critical role in assessing whether the reform influenced whether economic evaluations are used to a greater extent than in the past to address the issue of efficiency.

A first limitation of the present study concerns the fact that, even though conscious efforts were made to include healthcare decision-makers working at the macro-, meso-, and micro-level, it is uncertain if representatives from all stakeholders were included in the study. Another limitation is the risk of selection bias, as some participants were selected with the help of the Dutch National Healthcare Institute. This may have resulted in participants having a greater interest in the topic of this study than the average healthcare decision-maker, resulting in an overestimation of the actual use and knowledge of economic evaluations in the healthcare sector.

### Recommendations for research and practice

The use of economic evaluations in healthcare decision-making has the potential to improve the efficient use of resources, though this study suggests that the current use and impact of such studies is generally limited in the Dutch healthcare system. Therefore, strategies are needed to overcome the barriers that currently prevent economic evaluations from being used more extensively. Some preliminary recommendations as to how to overcome these barriers will be discussed below [26]. These recommendations are based on the present findings as well as relevant literature, but further research is needed to establish what recommendations will eventually be most effective in improving the uptake of economic evaluation in daily decision-making practice.

In order for economic evaluations to reach relevant decision-makers, it is essential to publish such studies in (non-scientific) journals and/or on websites that are easily accessible (e.g. open access journals). To provide healthcare decision-makers with relevant and ‘ready-to-use’ information, there may be value in developing a national database in which all relevant economic evaluations are collected and complemented by critical, easy-to-read summaries (e.g. a database comparable to the UK NHS-EED database) [27]. The addition of critical summaries is essential, as decision-makers often lack the time and skill set required to critically appraise economic evaluations [28]. Another possible means to improve the decision-makers’ economic evaluation skill set might be educating them about economic evaluation methods. Healthcare decision-makers may be educated through a variety of avenues, including the development of (online) handbooks and workshops, integrating economic evaluation methods into the medical curriculum, and by involving them in the commissioning and/or execution of studies [15, 25, 29]. To improve the perceived credibility of economic evaluations, guidelines are needed on how to conduct, report, and critically appraise such studies. Though several guidelines for the conduct and reporting of economic evaluations are already available (e.g. [9, 30, 31]), these could be supplemented with more user friendly guidelines designed specifically for practitioners. Another possible means to improve the use of economic evaluations is to establish a formal willingness-to-pay threshold for QALYs. Participants in this study were generally in favour of the introduction of such a threshold, but they rejected the idea of an explicit cut-off point. Instead, they were in favour of a bandwidth or categorization. Few of them, however, were willing to make a statement about the possible level of the threshold.

Participants also indicated a need for economic evaluations that are directly applicable to their decision-making context. Therefore, additional economic evaluations that are timely and relevant for healthcare decision-makers need to be performed. The fact that economic evaluations performed from the societal perspective are not directly applicable to a specific decision-making context may be overcome by using the two-perspective approach advocated by Brouwer et al. [32]. When using such an approach, economic evaluations are conducted from both the healthcare system and the societal perspective. In this way, economic evaluations are directly applicable to the healthcare sector, while simultaneously providing an indication of whether the “local perspective” of the healthcare sector is consistent with social optimality (i.e. societal welfare maximization) [32].

Incentives are needed for healthcare providers to provide care that is most likely to be cost-effective. One way to deal with the incentives to “over treat” may be to move away from a predominant “fee-for-service” system to a “pay-for-performance” system, in which healthcare providers receive a bonus if they meet or exceed certain agreed-upon quality or performance measures [33, 34].

Ethical considerations, such as the physicians’ responsibility to act in their patients’ best interest, may partially be dealt with by raising the physicians’ awareness of the fact that the provision of all possible treatment options, irrespective of their (cost-) effectiveness, might reduce the accessibility and quality of care to other clients and to Dutch society in general [35]. However, it is unrealistic to expect healthcare providers to make such decisions on their own [36, 37]. Therefore, identifications of cost-effective treatment options are best undertaken at the macro- and meso-level. This could take the form of narrowing medical indications and concurrently the definition of the basic healthcare package (at a macro-level), as well as developing clinical, best practice guidelines (at a meso-level).

Finally, strategies should be developed to overcome the public resistance to the use of economic evaluations in healthcare decision-making. Such strategies may include national campaigns (e.g. educating people about the importance of improving the efficiency of healthcare through the use of economic evaluations) as well as an increased transparency about the actual cost of treatment.

## Conclusion

Even though the use of economic evaluations in healthcare decision-making can potentially help decision-makers in allocating scarce resources as efficiently as possible, this study demonstrated that the current use and impact of economic evaluations in Dutch healthcare decision-making is limited at best. Therefore, strategies are needed to overcome the barriers that currently prevent economic evaluations from being used extensively.

## Additional file

Additional file 1: Topic list of the interviews. Description of data: Additional file 1 provides an overview of the semi-structured and structured questions of the interviews. (DOCX 14 kb)

## Abbreviations

CBA: Cost-benefit analysis
CEA: Cost-effectiveness analysis
CUA: Cost-utility analysis
HE: Health Economist
MA: Macro-level
ME: Meso-level
MI: Micro-level
n: Number
QALYs: Quality Adjusted Life Years
UK: United Kingdom
