# Supplementary material for: Economic evaluations of health technologies in Dutch healthcare decision-making: a qualitative study of the current and potential use, barriers, and facilitators
Source: BMC Health Serv Res. 2017 Jan 26;17:89. doi: 10.1186/s12913-017-1986-9 (PMC5270365; doi:10.1186/s12913-017-1986-9)
Supplement: Additional file 1: — Topic list of the interviews. Description of data: Additional file 1 provides an overview of the semi-structured and structured questions of the interviews. (DOCX 14 kb) [file 12913_2017_1986_MOESM1_ESM.docx]

**Additional file** 1**. Topic list of the interviews**

- Background of the participant

**Semi-structured questions**

- Current use of economic evaluations
  - Macro-level
  - Meso-level
  - Micro-level
- Factors influencing decision-making
  - Content of the basic insurance package
  - Implementation of innovations
  - Individual patient setting
- Opportunities for the use of economic evaluations
  - Opportunities and responsibilities of government agencies
  - Opportunities within the organisation
  - Opportunities in the individual patient setting
    - The physicians’ responsibility
- Barriers to the use of economic evaluations
  - Examples?
- Facilitators for the use of economic evaluations
  - How to implement?

**Structured questions**

- Knowledge about economic evaluations
  - What do you associate with the term ‘economic evaluation’?
  - Have you received training in economic evaluation-related topics?
    - Do you have a need for training in economic evaluation-related topics?
- Definitions of three economic evaluation designs (CEA, CBA, CUA)
  - CEA
    - Have you ever heard of the term “cost-effectiveness analysis”?
    - What does the term “cost effectiveness analysis” mean?
  - CUA
    - Have you ever heard of the term “cost-utility analysis”?
    - What does the term “cost utility analysis” mean?
  - CBA
    - Have you ever heard of the term “cost-benefit analysis”?
    - What does the term “cost benefit analysis” mean?
